# Tracheitis

**Published:** 1914-04

**Authors:** R. R. Daly

**Affiliations:** Atlanta, Ga.


Page(s) missing trachea and it must be treated locally. The treatment may be general in addition, but to get quick results, the applications should be local. It is well to remember that just touching the larynx and not getting into the trachea, itself, is in this regard, a general treatment.

In order to accomplish this, the patient needs further instruction as to his part of the "team work." In addition to teaching him how to hold his tongue with the napkin, he should be told what to expect. The application will always produce some spasm.

I tell my patient to be ready to do instantly exactly what I direct and rehearse him in advance in saying "hal." Then when I have reached the larynx with my applicator and the cords automatically close to bar further passage, I have him breathe or attempt to breath at the word of direction. Meantime, the applicator is resting on the cords. It takes only an instant, and when he begins to attempt to breathe, the applicator slips through and the tracheal region is under local treatment.

The great spasm that always comes at this instant should be foreseen and the alarm of the patient may be lessened by words of reassurrance.

If he is told to breathe through his nose, it will give him something else to think about and really warm and prepare the air better for its passage through the trachea. The excitement will soon be over but it is well always to warn the patient of this in advance, else he may think he has been roughly handled and fail to return.

Tracheitis may thus be treated with antiseptics and astringents and satisfactory results follow their application. The intratracheal application is necessary to promote a cure and general treatment will not do this. Such remedies as glycerine and ichthyol, nitrate of silver 1%, argyrol, petrogen iodine, saturated solution of iodoform in ether, etc., may be thus used with good results as they are indicated.

I have spoken thus in an elementary way, because in my judgment many of these cases have been overlooked through an impression that they are too difficult to observe and handle.

When there is tickling cough that can not be located either in the bronchi or larynx, the trachea should be examined and frequently, it will show the trouble. No expensive or unusual aparatus is necessary. A light, a head mirror, a laryngoscope and a long curved applicator with some cotton to twist firmly upon it and one is fully equipped.

